# Supplementary material for: The development of lower-atmosphere turbulence early in a solar flare
Source: Sci Adv. 2018 Dec 5;4(12):eaav2794. doi: 10.1126/sciadv.aav2794 (PMC6281430; doi:10.1126/sciadv.aav2794)
Supplement: http://advances.sciencemag.org/cgi/content/full/4/12/eaav2794/DC1 [file supp_4_12_eaav2794__index.html]

Science Advances | Science Advances

## Supplementary Materials

**The PDF file includes:**

- Fig. S1. A context image of the solar flare in active region 12615.
- Fig. S2. Si IV contribution function.
- Fig. S3. A comparison of Si IV 1402.77 Å and Mg II 2796.35 Å centroid positions.
- Fig. S4. Temporal evolution of Si IV 1402.77 Å line properties during the flare (for two individual pixels).

Download PDF

**Other Supplementary Material for this manuscript includes the following:**

- Movie S1 (.mp4 format). Light curves and the images and line spectra of flare SOL2016-12-06T10:36:58 at all studied times during the flare rise, peak, and decay (associated with Fig. 1).
- Movie S2 (.mp4 format). Plasma velocity fluctuations in space and time due to the passage of a single wave (associated with Fig. 4).
- Movie S3 (.mp4 format). Plasma velocity fluctuations in space and time due to the passage of multiple interacting waves (associated with Fig. 4).

**Files in this Data Supplement:**

- Adobe PDF - aav2794\_SM.pdf
