# Supplementary material for: The development of lower-atmosphere turbulence early in a solar flare
Source: Sci Adv. 2018 Dec 5;4(12):eaav2794. doi: 10.1126/sciadv.aav2794 (PMC6281430; doi:10.1126/sciadv.aav2794)
Supplement: http://advances.sciencemag.org/cgi/content/full/4/12/eaav2794/DC1 [file aav2794_SM.pdf]

Science Advances
AAAS

advances.sciencemag.org/cgi/content/full/4/12/eaav2794/DC1

Supplementary Materials for

The development of lower-atmosphere turbulence early in a solar flare

N. L. S. Jeffrey*, L. Fletcher, N. Labrosse, P. J. A. Simões

*Corresponding author. Email: natasha.jeffrey@glasgow.ac.uk

Published 5 December 2018, *Sci. Adv.* **4**, eaav2794 (2018)
DOI: 10.1126/sciadv.aav2794

The PDF file includes:

Fig. S1. A context image of the solar flare in active region 12615.
Fig. S2. Si IV contribution function.
Fig. S3. A comparison of Si IV 1402.77 Å and Mg II 2796.35 Å centroid positions.
Fig. S4. Temporal evolution of Si IV 1402.77 Å line properties during the flare (for two individual pixels).

Other Supplementary Material for this manuscript includes the following:

(available at advances.sciencemag.org/cgi/content/full/4/12/eaav2794/DC1)

Movie S1 (.mp4 format). Light curves and the images and line spectra of flare SOL2016-12-06T10:36:58 at all studied times during the flare rise, peak, and decay (associated with Fig. 1).
Movie S2 (.mp4 format). Plasma velocity fluctuations in space and time due to the passage of a single wave (associated with Fig. 4).
Movie S3 (.mp4 format). Plasma velocity fluctuations in space and time due to the passage of multiple interacting waves (associated with Fig. 4).

Supplementary Materials

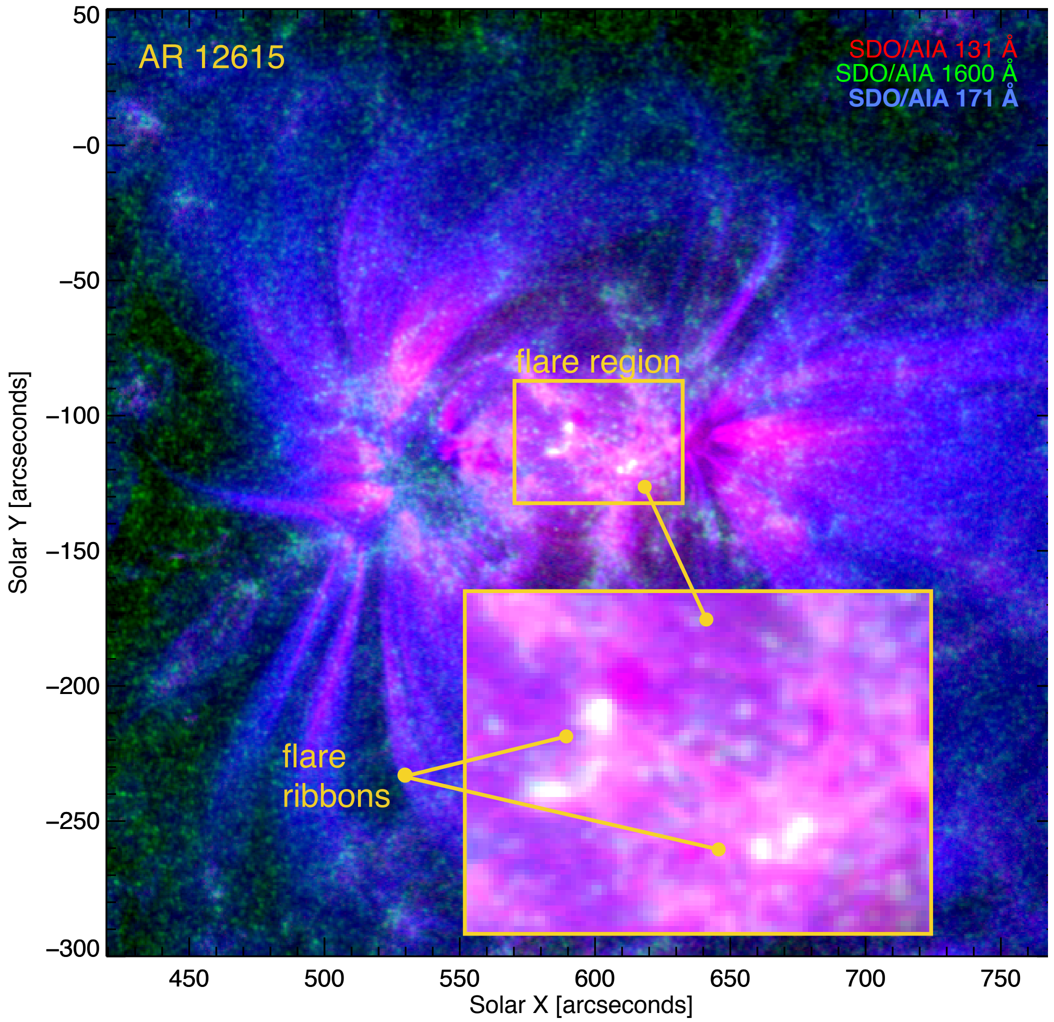

Fig. S1. A context image of the solar flare in active region 12615 using a composite Solar Dynamics Observatory (SDO) Atmospheric Imaging Assembly (AIA) image using wavelengths of 131 Å, 1600 Å and 171 Å. Zooming in on the flare region clearly shows the flare ribbons.

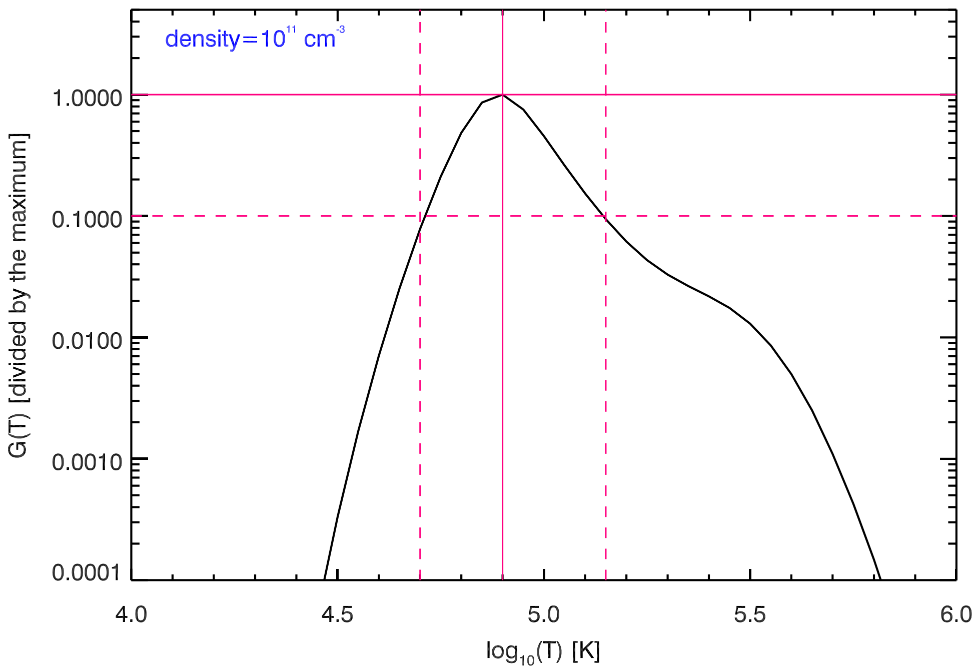

Fig. S2. Si IV contribution function G(T) showing that the emission of Si IV peaks close to log T=4.9~80000 K. Over a temperature range of log T~4.7-5.15 (50000-140000) K, the emission of Si IV falls by one order of magnitude. Si IV is most likely formed close to its peak formation temperature of 80000 K, but it can be formed over a range of temperatures.

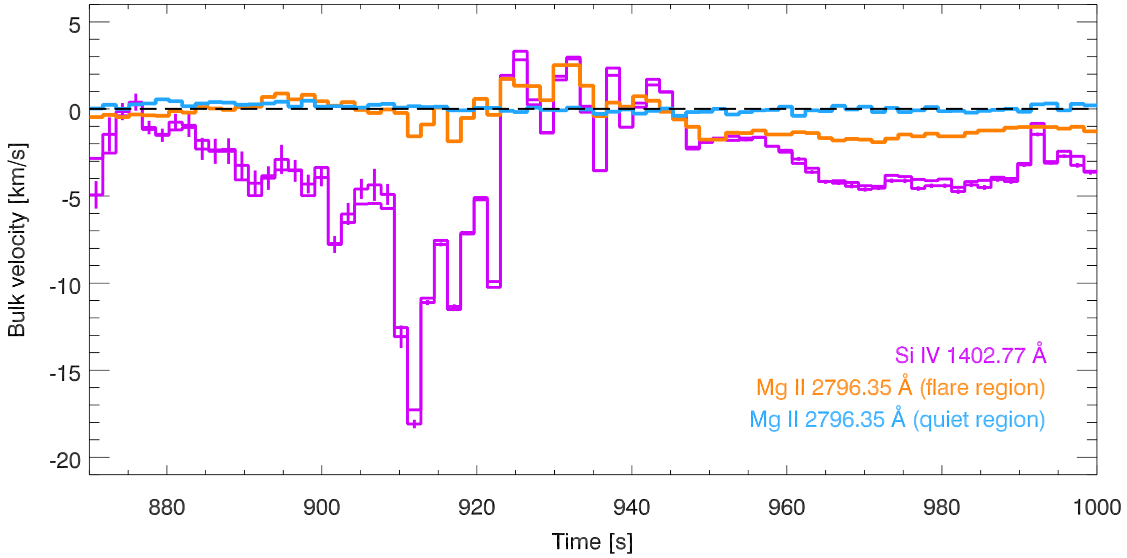

Fig. S3. A comparison of Si IV 1402.77 Å and Mg II 2796.35 Å centroid positions. A comparison of Si IV 1402.77 Å (purple) and Mg II 2796.35 Å (orange) centroid positions (as bulk velocities) with time. The Mg II 2796.35 Å line shows similar velocity movements to the Si IV 1402.77 Å line, but with smaller values. We can see that no sharp jumps in the centroid position are observed in the Mg II 2796.35 Å quiet region (blue).

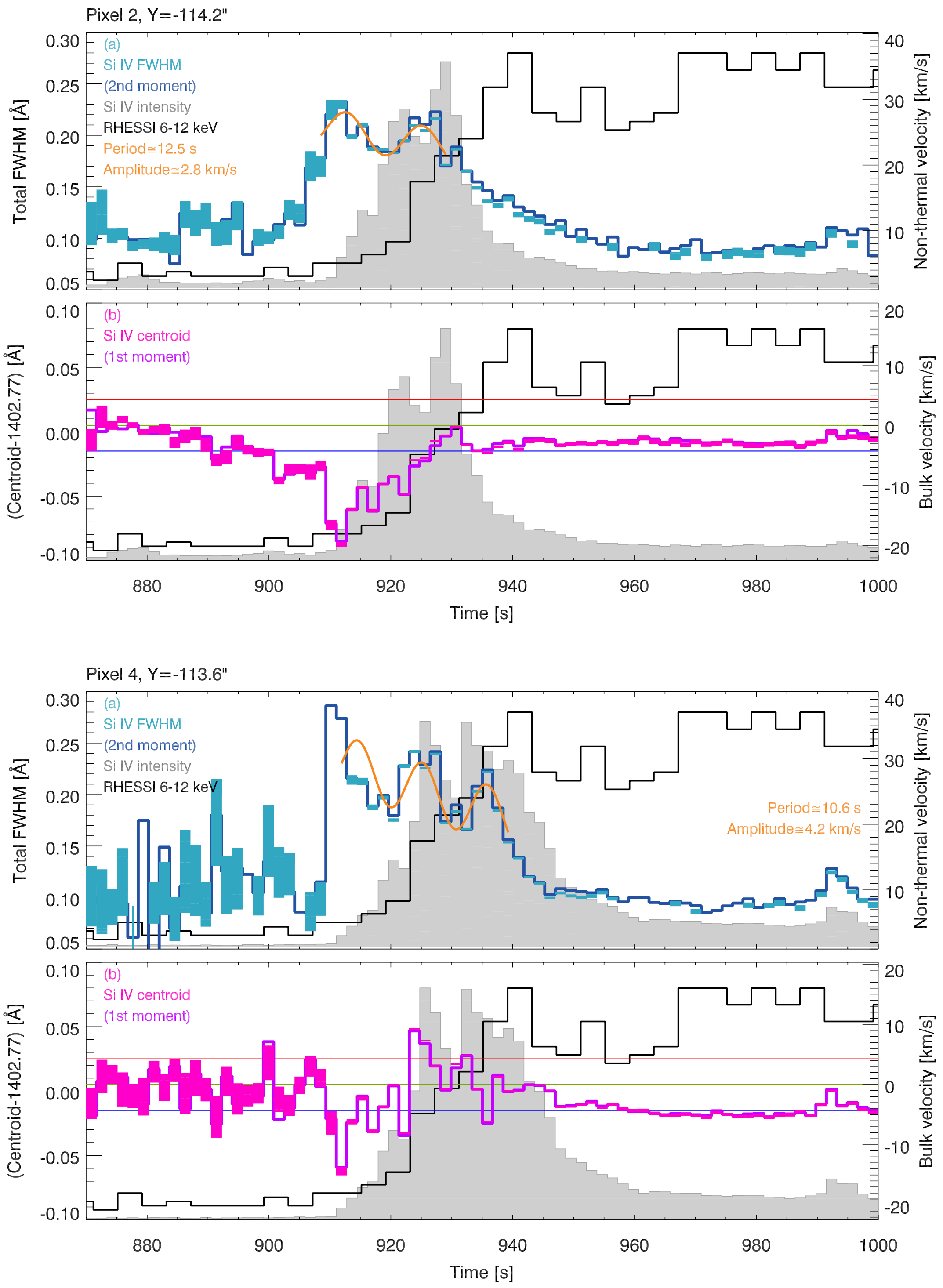

Fig. S4. Temporal evolution of Si IV 1402.77 Å line properties during the flare (for two individual pixels). (a) the total FWHMs and non-thermal velocities v_{nth} (turquoise), and (b) the centroid positions and bulk velocities v (purple), for two individual pixels: pixel 2 at Y=-114.2” (top two panels) and pixel 4 at Y=-113.6” (bottom two panels). Both the results of Gaussian fitting and the moments analysis are shown (see legend) and both give near identical results. The Si IV integrated intensity (grey), and the RHESSI 6-12 keV light curve (black), are also displayed. A sinusoidal function is fitted to the variations in v_{nth}, estimating the period P, and amplitude A. The inferred reference wavelength is shown (lime line) ± uncertainty (red, blue lines).
